# Harnessing Light Wavelengths to Enrich Health-Promoting Molecules in Tomato Fruits

**DOI:** 10.3390/ijms26125712

**Published:** 2025-06-14

**Authors:** Bruno Hay Mele, Ermenegilda Vitale, Violeta Velikova, Tsonko Tsonev, Carolina Fontanarosa, Michele Spinelli, Angela Amoresano, Carmen Arena

**Affiliations:** 1 Department of Biology, University of Naples Federico II, Via Cinthia, 80126 Napoli, Italy; ermenegilda.vitale@unina.it (E.V.); c.arena@unina.it (C.A.); 2 NBFC—National Biodiversity Future Center, 90133 Palermo, Italy; 3 Institute of Plant Physiology and Genetics, Bulgarian Academy of Sciences, Acad. G. Bonchev Street, Bldg. 21, 1113 Sofia, Bulgaria; violet@bio21.bas.bg (V.V.);; 4 Department of Chemical Sciences, University of Naples Federico II, Via Cinthia, 80126 Napoli, Italy

**Keywords:** antioxidants, biofortification, functional food, light quality, phenolic compounds, tomato, human health

## Abstract

The tomato (*Solanum lycopersicum* L.) is one of the most consumed crops worldwide and a source of antioxidants. Given the role the latter play against oxidative stress and free radical-related diseases, enhancing tomato bioactive compound production would be appealing for a wide range of applications in the fields of nutrition, pharmacy, and biotechnology. This study explores a sustainable and innovative approach: the modulation of specific light spectra to boost the production of bioactive compounds in tomatoes (cultivar ‘Microtom’). We investigated how three light regimes—white fluorescent (FL), full-spectrum (FS), and red-blue (RB)—influence the accumulation of polyphenols and other key nutraceuticals during plant growth. Our findings reveal that full-spectrum (FS) light significantly enhances the levels of polyphenols, flavonoids, tannins, ascorbic acid, and lycopene in tomato fruits, compared to those grown under RB or FL light. Interestingly, fruits from RB light-grown plants showed the highest carotenoid concentrations and antioxidant capacity. These results suggest that light quality actively modulates the expression of key enzymes in the phenylpropanoid and flavonoid biosynthetic pathways, shaping each fruit’s unique metabolic fingerprint. Cluster analysis confirmed that RB, FL, and FS conditions lead to distinct polyphenolic profiles, each with notable health-promoting potential. Our results highlight a promising avenue: tailoring light environments to enhance the functional value of crops, bridging agriculture, nutrition, and biomedicine in a sustainable way.

## 1. Introduction

Tomatoes (*Solanum lycopersicum* L.) are among the most widely consumed horticultural crops. They are valued for their culinary versatility and for their rich content of bioactive compounds with significant health benefits [1,2]. Key phytochemicals found in tomatoes—including lycopene, flavonoids, phenolic acids, and vitamin C—have been linked to antioxidant activity, reduced risk of chronic diseases, and overall improved human health [3,4].

Tomato fruits are rich in various phenolic compounds, including quercetin, kaempferol, naringenin, lutein, and caffeic, ferulic, and chlorogenic acids. The concentrations of these compounds are linked to the prevention of hypertension, oxidative stress, and cardiovascular diseases [4].

Given their health-promoting effects, there is growing interest in eco-friendly strategies to enhance the synthesis of specific bioactive compounds in this crop. However, this is a complex challenge, since the synthesis and accumulation of any antioxidant compound involves intricate metabolic processes. Different strategies can be employed to achieve this purpose, and one of the most effective and sustainable is the light-dependent regulation.

Recent advances in controlled-environment agriculture have highlighted the crucial role of light quality in influencing plant secondary metabolism. Light serves not only as an energy source for photosynthesis but also as an important environmental signal that affects gene expression and biochemical pathways. The development of light-emitting diode (LED) technology, which enables precise spectrum modulation, offers a unique opportunity to precisely regulate various aspects of plant development. By exposing plants to specific light quality regimens, it is possible to modulate photosynthesis, increase the shelf life of plants and fruits, and induce accumulation of phytochemicals [5,6,7,8,9,10]. A crucial point to consider is that the effects of light quality on the modulation of secondary metabolites are specific to each plant species. Consequently, the benefits observed in one species after exposure to a specific regimen may be difficult or impossible to replicate in another [9].

In tomatoes and other plant species, light wavelengths spanning from ultraviolet to far-red light act as crucial regulatory signals. These signals are detected by specific photoreceptors: phytochromes (which respond to red and far-red radiation), cryptochromes (which respond to blue and UV-A radiation), and phototropins (which detect blue radiation). The signal perception by these photoreceptors initiates specific metabolic and developmental responses that contribute to the regulation of plant growth and adaptation [10,11].

For example, cryptochromes play a crucial role in mediating changes in gene expression induced by blue light, particularly in the regulation of flowering and the accumulation of phenolic compounds, especially flavonoids like rutin [10]. Research indicates that blue light enhances the activity of key enzymes in the shikimate and phenylpropanoid pathways, including phenylalanine ammonia lyase (PAL), chalcone synthase (CHS), and chalcone isomerase (CHI), as well as the activity of enzymes involved in flavonoid synthesis, e.g., flavonol synthase (FLS) [7].

Furthermore, molecular and functional characterization of the tomato cryptochrome gene family has shown that the cry1a gene regulates seedling photomorphogenesis, anthocyanin accumulation, and the development of adult plants; however, it does not influence flowering time or fruit pigmentation [12].

Recent research has also demonstrated that supplying fruits with red and blue lights for three hours in the morning can successfully increase the content of six phenolic acids and one flavonoid (p-hydroxybenzoic acid, caffeic acid, cynarin, cinnamic acid, benzoic acid, ferulic acid, and quercetin). Conversely, this treatment reduces the amounts of three phenolic acids and one flavonoid (gentisic acid, 4-coumaric acid, gallic acid, and rutin) [13].

Fruit-localized phytochromes promote the production and accumulation of lycopene. Specifically, phytochrome B2 has been reported to positively regulate the pigmentation of mature green fruits [12]. Red light, along with different light intensities, influence the biosynthesis of carotenoids by promoting the initial step of carotenogenesis, and, particularly, modulating phytoene synthesis activity, which is a key control stage of carotene biosynthesis [14].

Additionally, the combined application of red and blue light boosts the synthesis of lycopene and β-carotene, inducing an earlier ripening in tomato fruit [6].

Continuous exposure to blue light has proven to increase the levels of free amino acids in post-harvest tomato fruits [13]. Furthermore, the combination of red and blue light (with a ratio of 3:1) induced higher levels of fructose, glucose, and sucrose by promoting the accumulation of proteins involved in glucose metabolic pathways [13]. Conversely, excessive exposure to long far-red (FR) light can lead to a decrease in soluble sugar content in ripe tomato fruits, while blue light is recognized for significantly prolonging the shelf life of tomatoes and maintaining the firmness of the fruit by slowing down the ripening process [6].

A new open research field is how selected light wavelengths may condition the polyphenol profile of plant edible tissues, including fruits [9].

Although previous research has elucidated the effect of discrete wavelengths on metabolite accumulation in tomatoes, the influence of broader and more complex spectral compositions remains insufficiently characterized. Advancing our understanding in this area could facilitate the targeted enhancement of antioxidant compounds, enabling the fine-tuning of fruit quality in accordance with specific metabolic profiles. To address this knowledge gap, we examined the effects of three customized light quality regimes—fluorescent (FL), full spectrum (FS), and red-blue (RB)—on the concentration of health-promoting compounds in tomato fruits. Our focus was on the dwarf tomato cultivar ‘Microtom’, which is known for its compact size, rapid growth, and short life cycle. Based on these characteristics, Microtom is particularly well-suited for high-density cultivation and offer a unique opportunity to boost antioxidant content more efficiently than traditional tomato varieties [8]. In this paper, we aimed to explore whether specific wavelengths, differing across light quality regimens, modulate the accumulation of key secondary metabolites in Microtom, and investigate the polyphenol profiles associated with the three light regimens. The findings may deepen insights into the relationship between light quality and tomato fruit metabolites exploring the opportunities to harness tomato-derived molecules for biochemical, nutraceutical, and medical purposes.

By characterizing the effects of specific light spectra on tomato secondary metabolism, this study provides a foundation for improving the nutritional quality of Microtom fruits through targeted light treatments, with potential applications in functional food production and sustainable agriculture.

## 2. Results

### 2.1. Influence of Light Quality Regimes on Biochemical Markers

**Figure 1:**
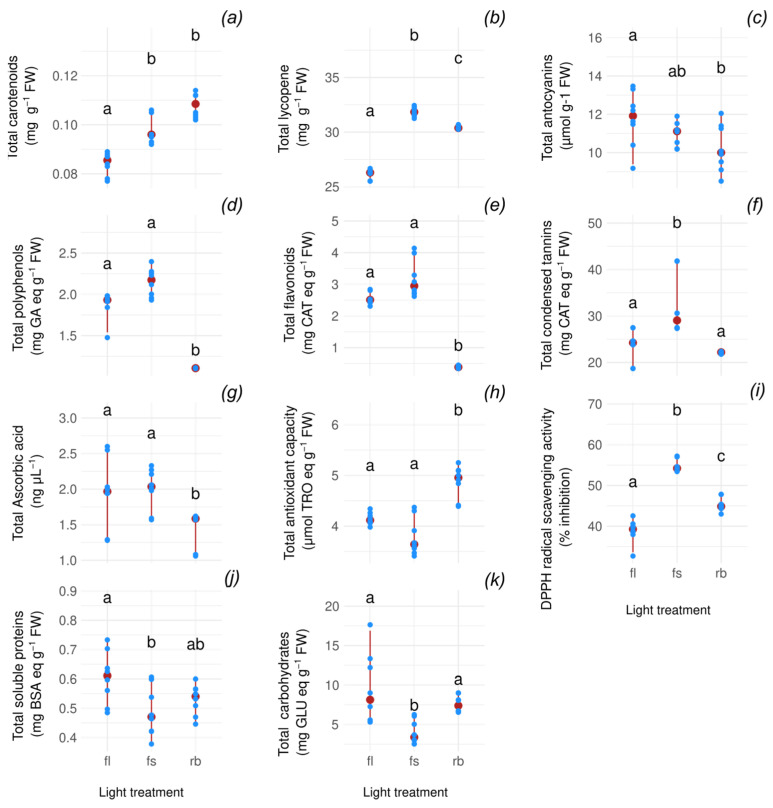
Boxplots of biochemical marker levels across light treatments. Each facet displays a different marker, with individual sample points overlaid and medians highlighted in red. (**a**) total carotenoids, (**b**) total lycopene, (**c**) total anthocyanins, (**d**) total polyphenols, (**e**) total flavonoids, (**f**) total condensed tannins, (**g**) total ascorbic acid, (**h**) total antioxidant capacity, (**i**) DPPH radical scavenging activity, (**j**) total soluble proteins, (**k**) total carbohydrates. Letters indicate statistically significant groupings based on Dunn’s post hoc test following a Kruskal–Wallis analysis (*p* adjusted via Holm correction).

**Figure 2:**
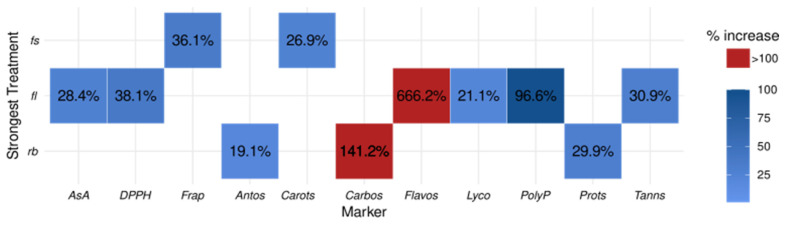
Heatmap summarizing the strongest treatment effects on biochemical markers. Tile color indicates percent change between the strongest and weakest treatment groups per marker, with values annotated. Only statistically significant differences (Dunn’s test, Holm-adjusted *p* < 0.05) are shown. AsA: Ascorbic Acid; DPPH: DPPH scavenging activity; FRAP: total antioxidant capacity; Antos: total anthocyanins; Carots: total carotenoids; Carbos: total carbohydrates; Flavos: total flavonoids; Lyco: total lycopene; PolyP: total polyphenols; Prots: total soluble proteins; Tanns: total condensed tannins.

Tomatoes grown under full-spectrum (FS) and red-blue (RB) light regimes exhibited significantly higher carotenoid (adj.*p* ≤ 2.27 × 10^−2^ and adj.*p* ≤ 5.87 × 10^−5^ for FS and RB, respectively) and lycopene (adj.*p* ≤ 1.79 × 10^−5^ adj.*p* ≤ 4.72 × 10^−2^ for FS and RB, respectively; adj.*p* ≤ 4.72 × 10^−2^ for FS vs. RB) concentrations compared to those under fluorescent light (FL) [Figure 1a,b]. The RB regime showed the most pronounced effect, with a 26.9% increase in lycopene relative to FL, while FS treatment enhanced lycopene levels by 21.1% [Figure 2]. In contrast, total anthocyanins (adj.*p* ≤ 4.41 × 10^−2^ for FL vs. RB), polyphenols (adj.*p* ≤ 2.66 × 10^−2^ and adj.*p* ≤ 4.80 × 10^−5^ for FL and FS, respectively), and flavonoids (adj.*p* ≤ 2.40 × 10^−2^ and adj.*p* ≤ 5.63 × 10^−5^ for FL and FS, respectively) decreased markedly under RB light [Figure 1c–e]. Anthocyanin content was 19.1% lower than FL-grown tomatoes, while polyphenols and flavonoids decreased by 96.6% and 666.2%, respectively, compared to FS-treated fruits [Figure 2]. Conversely, condensed tannins were more abundant in FS tomatoes (adj.*p* ≤ 2.18 × 10^−2^ and adj.*p* ≤ 4.02 × 10^−4^ for FL and RB, respectively), surpassing RB fruits by 30.9% [Figure 1f and Figure 2]. Ascorbic acid concentration decreased significantly in RB tomatoes (adj.*p* ≤ 2.63 × 10^−2^), showing a 28.4% reduction compared to FL [Figure 1g and Figure 2]. The total antioxidant capacity and antioxidant activity displayed divergent trends: the FS regime yielded the lowest total antioxidant capacity (36.1% below RB) and the highest antioxidant activity (38.1% above FL) (capacity: adj.*p* ≤ 1.04 × 10^−2^ for FL vs. RB, adj.*p* ≤ 1.93 × 10^−4^ for FS vs. RB; activity: adj.*p* ≤ 1.78 × 10^−5^ for FLvs FS, adj.*p* ≤ 4.71 × 10^−2^ for FL vs. RB, adj.*p* ≤ 4.71 × 10^−2^ for FS vs. RB) [Figure 1h,i and Figure 2]. Finally, soluble proteins (adj.*p* ≤ 3.99 × 10^−2^) and carbohydrates (adj.*p* ≤ 5.90 × 10^−3^ for FS vs. FL; adj.*p* ≤ 5.90 × 10^−3^ for FS vs. RB) displayed the same trend with the highest amount detected in FL fruits, 29.9% and 141.2%, respectively, higher than FS ones [Figure 1j,k]. All analyzed markers showed an average increment of 28.8% in the strongest treatments compared to the weakest ones [Table 1, Figure 2], except for the carbohydrates (141.2%), polyphenols (99%), and flavonoids (666.2%) that stood out as the most significantly affected markers, demonstrating a pronounced response to light quality treatments.

### 2.2. Correlation Between Light Wavelengths and Biochemical Markers

**Figure 3:**
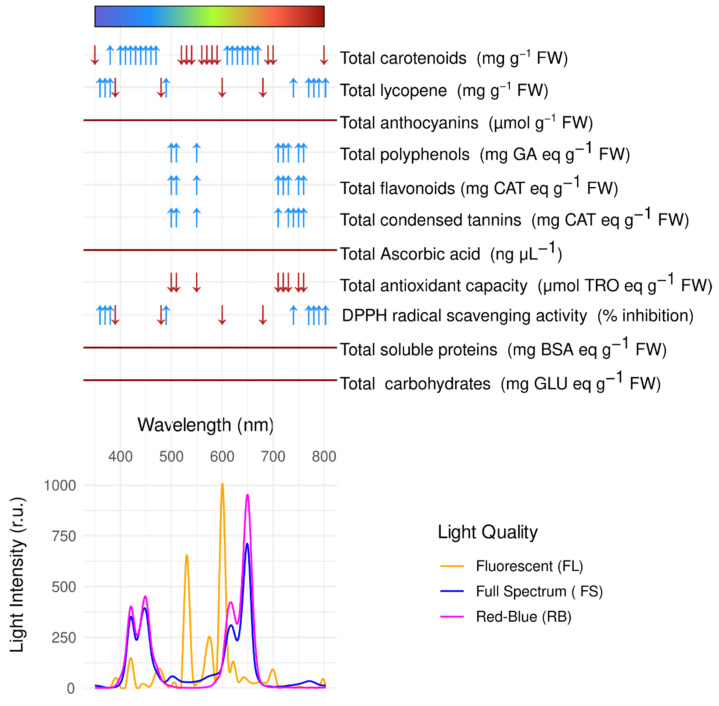
Spectral reflectance curves for each sample, grouped by treatment. Arrows indicate wavelengths that are significantly correlated with biochemical marker levels (FDR-adjusted *p* < 0.05; Spearman correlation > 0.75). Upward and downward arrows represent strong positive and negative correlations, respectively. Solid lines indicate no significant correlation.

As reported in Figure 3, anthocyanins, ascorbic acids, proteins, and carbohydrates did not show any significant correlation with wavelengths of the three light quality regimes.

Although pairwise correlations between markers were not statistically significant [Figure S1], several markers exhibited similar correlation patterns with light intensity across the spectrum, suggesting that these markers may respond to shared light-driven mechanisms rather than to intrinsic interdependence. This pattern underscores the importance of interpreting correlation coefficients alongside their confidence intervals, especially in cases where biological variability or sample size may affect estimate precision. While some correlations are statistically significant, wide intervals reflect underlying uncertainty and suggest that these associations should be validated in independent datasets or through targeted experiments.

The total carotenoids are boosted by wavelengths within the blue ([400–470] nm, ρ = 0.89, adj.*p* ≤ 1.03 × 10^−8^) and orange/red bands ([610–670] nm, ρ = 0.89, adj.*p* ≤ 1.03 × 10^−8^), while reductions are induced by wavelength within the 520–590 nm range (ρ = −0.89, adj.*p* ≤ 1.03 × 10^−8^). Within the carotenoid group, lycopene production is stimulated by violet ([360–380] nm, ρ = 0.94, adj.*p* ≤ 1.99 × 10^−11^) and far red ([740–790] nm, ρ = 0.94, adj.*p* ≤ 1.99 × 10^−11^) and reduced by blue light ([400–470] nm, ρ = −0.94, adj.*p* ≤ 1.99 × 10^−11^). The antioxidant activity follows the same trend as lycopene. Polyphenols, flavonoids, and tannins increase simultaneously at two wavelength groups: green ((500,510,550); ρ = −0.90, adj.*p* ≤ 1.51 × 10^−8^) and far red ([710, 760] (ρ = −0.90, adj.*p* ≤ 1.51 × 10^−8^). Antioxidant capacity showed a decrease in green ([500–550]) and far-red ([710–760]) ranges. In this case, despite a highly significant correlation (ρ = −0.83, adj.*p* ≤ 2.93 × 10^−6^), the wide confidence interval [−0.96, −0.45] indicates uncertainty in the estimate.

### 2.3. Cluster Analysis of the Polyphenolic Profile

**Figure 4:**
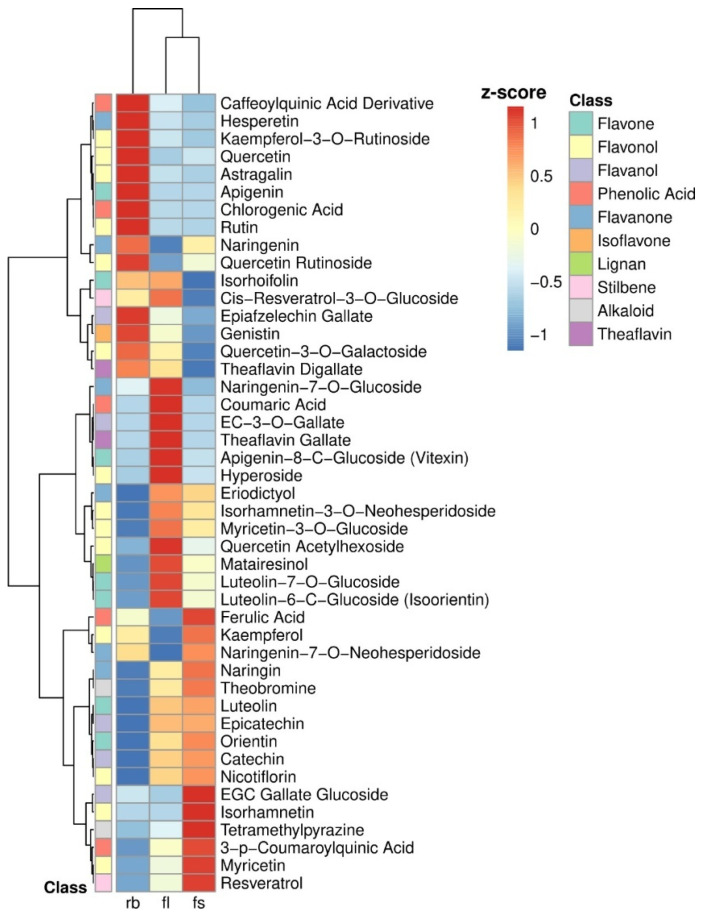
Hierarchical clustering heatmap of median-centered, scaled polyphenol profiles across light treatments. Rows represent individual polyphenols, and columns represent treatment groups. Clustering was performed using Manhattan distance and Ward’s linkage method to reveal treatment-driven patterns in metabolite abundance. A color gradient scale for Z-scores (mean-centered and standard deviation-scaled values) and dendrogram branch lengths are included to aid interpretation.

A hierarchical clustering heatmap [Figure 4] clearly separated RB fruits, in the first cluster, from FL and FS fruits, grouped in the second cluster. The heatmap pointed out the highest amount of caffeoylquinic acid derivative, hesperetin, kaempferol-3-O-Rutinoside, quercetin, astragalin, apigenin, chlorogenic acid, rutin, naringenin, quercetin rutinoside, epiafzelechin gallate, genistin, quercitin-3-O-galactoside, theaflavin digallate in RB tomatoes; Naringenin-7-O-glucoside, coumaric acid, EC-3-O-gallate, theaflavin gallate, apigenin-8-C-glucoside, hyperoside, eriodictyol, isorhamnetin, myricetin, quercetin acetylhexoside, matairesinol, luteolin in FL tomatoes; and, ferulic acid, kaempferol, naringenin-7-O-neohesperidoside, naringin, theobromine, luteolin, epicatechin, orientin, catechin, nicotiflorin, EGC gallate glucoside, isorhamanetin, tetramethylpyrazine, 3-p-coumaroylquinic acid, myricetin, resveratrol in FS tomatoes.

## 3. Discussion

The results of this study demonstrate that different light spectral compositions can distinctly modulate the accumulation of bioactive compounds in tomato fruits during growth, leading to treatment-specific metabolic profiles in fruits.

Ascorbic acid is one of the most important antioxidants found in tomatoes, and its synthesis is particularly sensitive to light intensity and quality [8]. Pure blue or dichromatic blue-red light typically stimulates AsA production in tomato fruits coming from plants grown under white, red, or green light [7,15]. According to results reported by Vitale et al. [8], we observed the lowest AsA concentration in RB and the highest in FL and FS fruits. Since no significant pairwise correlation was found between individual wavelength and AsA concentration, we concluded that the effect is not wavelength specific. However, a consistent difference in AsA concentration was observed among treatments, with the RB condition resulting in significantly lower median AsA levels due to the limited amount of far-red light (less than 1/3rd and 1/10th of FS and FL at 800 nm, respectively). Supporting this idea, previous studies have shown that storing red tomatoes under monochromatic Far-Red Light can increase AsA levels [16].

Far-red light plays a significant role in the biosynthesis of carotenoids and lycopene through phytochrome-mediated signaling pathways. During the fruit ripening process, from the immature green stage to the mature red stage, red and far-red wavelengths penetrate the epidermis and pericarp of immature fruits. Therefore, the activation of phytochromes localized in the fruit is promoted, driving the transition of chloroplasts to chromoplasts alongside the breaking down of chlorophylls, which preferentially absorbs red light.

This process enhances carotenoid biosynthesis in the fruit pericarp and the turning to the red-ripe stage. However, while phytochromes have been detected in the epidermis and pericarp, there is currently no evidence regarding their tissue-specific distribution within tomato fruits [14,16,17].

In ripe red tomatoes, the primary compounds responsible for the red color of the fruits are carotenoids, specifically lutein (1–5%), β-carotene (5–10%), and lycopene (approximately 90%). Both lycopene and polyphenol concentration strongly respond to variation in the light quality, and, in particular, blue wavelengths and UV, perceived by cryptochromes [12]. Moreover, it has been demonstrated that UV irradiation enhances the content of carotenoids, specifically lycopene and β-carotene, in tomato fruits [18,19]. Accordingly, carotenoids were more abundant in FS and RB tomatoes exposed to higher proportions of blue (400–470 nm) and orange-red lights (610–670 nm), showing positive correlations with these wavelength ranges, crucial for the activation of plant photoreceptors. Lycopene, however, showed an interesting pattern: it is correlated positively with far-red (470–790 nm) and violet (360–380 nm) wavelengths but negatively with blue light. The positive correlation with far-red is supported by the phytochrome-mediated pathway, which emphasizes fruit development. Conversely, violet light may trigger specific responses that enhance lycopene production as an antioxidant. The negative correlation with blue light suggests a complex regulatory mechanism in which blue light signaling pathways can inhibit lycopene synthesis under certain conditions.

Therefore, since lycopene was more abundant in FS fruits, it is likely to suppose that the full spectrum (FS) regime boosts its production due to the higher proportion of far-red (4.13%) and violet-blue (37.64%) amount [5]. In contrast, the lower lycopene levels in FL tomatoes may be ascribed to the reduced contributions of far-red (2.21%), blue (12.4%), and violet (0.6%) wavelengths, considering that no difference in red wavelength occurred between FL and FS treatments (45,48% vs. 48.79, respectively). A possible explanation of the decrease in lycopene concentration in RB fruits is the absence of violet/far-red wavelengths despite the presence of 60% red and 40% blue in this light regime. Further research is needed to elucidate the precise molecular mechanisms underlying the wavelength-specific effects on lycopene biosynthesis.

Total protein and carbohydrate concentrations in fruits under different light quality regimes depend on plant photosynthetic activity. Vitale et al. [20] demonstrated that RB light treatment enhances photosynthesis in Microtom plants by favoring the stomata opening particularly responsive to blue light wavelengths. The higher photosynthetic rates promote nitrogen assimilation and nitrate metabolism [21], as well as soluble protein synthesis [22]. In particular, blue light boosts the aminoacidic concentration in fruits [7,13,23]. Our study showed that while RB and FL fruits had similar concentrations of soluble proteins, FL fruits contained a higher protein content than FS fruits. This difference may be attributed to the greater amount of green light present in the FS regime (25%) compared to FL (6.57%) and RB (0%) ones. Since green light penetrates deeper into the leaf than either red or blue light, it can enhance photosynthesis. This, in turn, influences nitrogen metabolism, leading to increased protein levels [8,20].

Soluble carbohydrates, like total proteins, are extremely responsive to light spectrum modulation during plant growth [7]. In our study, carbohydrate concentrations were higher in FL and RB compared to FS fruits, indicating a strong influence of light quality on sugar metabolic pathways. Specifically, the elevated content of carbohydrate in RB plants may be due to the highest percentage of blue wavelengths in this treatment. Sugar accumulation may derive from the overexpression of blue light photoreceptor CRY1 and CRY2 [12,24], elevated photosynthesis resulting from greater stomatal opening [7,8], and higher sucrose metabolism enzyme (sucrose synthase, invertases) activity [7,25]. These factors increase sucrose transport into the fruits, contributing to the rise in fructose, glucose, or starch under blue light. The combination of blue and red wavelengths also promotes glucose metabolic pathway [7,13]. In contrast, the lower carbohydrate amount observed under FS regime may be related to the reduced amount of red and blue wavelengths and the presence of far-red, which has been demonstrated to decrease soluble sugar level in ripe tomatoes [6]. Interestingly, under the FL regime, the lower fractions of red and blue lights may have been compensated by the combined application of green and orange percentages. These specific wavelengths enhance photosynthesis, and improve water use efficiency, positively influencing the translocation of photoassimilates from the leaves to the fruits [8,23,26].

Previous studies on tomato fruits have shown that exposure to red and blue wavelengths stimulates the accumulation of polyphenols. These compounds may exhibit variable patterns during ripening—increasing, decreasing, or remaining stable—contributing to enhance the antioxidant capacity and reactive oxygen species (ROS) removing [2,14,18,27]. The rise in antioxidant levels is likely due to a parallel increase in polyphenols, including flavonoids and carotenoids, particularly lycopene [8,12,28]. In the polyphenol modulation, the role of green and far-red wavelengths is particularly worthy of attention. In our study, we found that green and far-red portions were positively correlated with higher levels of polyphenols, flavonoids, and tannins, which contribute to the overall antioxidant activity of the fruits. Interestingly, previous research demonstrated that storing tomatoes under green light combined with red or far-red light can enhance both phenolic content and total antioxidant capacity [29,30].

Interestingly, the trends observed for total antioxidant capacity (FRAP) and radical scavenging activity (DPPH) did not fully align, suggesting that these assays describe different aspects of the antioxidant power of Microtom fruits. This discrepancy may be attributed to diverse causes: different chemical principles and reaction mechanisms proper of each assay (radical scavenging for the DPPH versus electron transfer/reducing power for FRAP), the structural diversity of the compounds which is better expressed in one assay over the other, the light quality-dependent modulation of the antioxidant profiles, which may enhance compounds favoring either radical scavenging or reducing power. Indeed, the effectiveness of polyphenolic compounds, including tannins, flavonoids, and anthocyanins, as free radical scavengers may differ considering their structural characteristics, such as proton and electron-donating ability, number and position of hydroxyl groups, presence of glycosylation, and arrangement of carboxylate and hydroxyl groups on the phenolic ring. Some phenolics are more effective in reducing ferric ions, as measured by FRAP assays, while others function better as hydrogen donors for radical scavenging, which is assessed by DPPH assays [9]. Consequently, the variation in phenolic profiles observed under FL, FS and RB light regimes, likely contribute to the differences between FRAP and DPPH results. Similarly, other antioxidants such as ascorbic acid, carotenoids (including lycopene), also respond differently to light quality and influence the assays in different ways. Their structural features determine whether they act as electron or hydrogen donors, further contributing to the assay discrepancies. However, further target analysis would be necessary to definitively elucidate the biochemical basis for this divergence.

The hierarchical clustering heatmap clearly differentiated RB fruits from FL and FS fruits, highlighting distinct profiles of phenolic and flavonoid compounds. RB tomatoes showed high concentrations of metabolites such as caffeoylquinic acid derivatives, hesperetin, kaempferol-3-O-rutinoside, quercetin, rutin, and theaflavin digallate—compounds known for their antioxidant, anti-inflammatory, and potentially chemopreventive properties [Table S1]. In contrast, FL and FS fruits displayed different metabolic patterns, with FL being rich in glycosylated flavonoids (such as naringenin-7-O-glucoside and apigenin-8-C-glucoside), and FS containing phenolic compounds like ferulic acid, catechin, and resveratrol.

Our data demonstrated that these differences are due to the influence of light quality experienced by the plants during growth [9]. Light is one of the main environmental signals regulating gene expression involved in the biosynthesis of secondary metabolites, including polyphenols [31]. Blue and UV-A light are known to stimulate the production of flavonoids and phenylpropanoids by activating key enzymes in the phenolic metabolism pathway. Red and far-red light, on the other hand, can modulate the accumulation of compounds related to ripening and oxidative stress responses [32].

The enrichment of RB fruits in high-value nutraceutical compounds is likely linked to more favorable light conditions for synthesizing these metabolites, such as a specific red-to-blue light ratio. Conversely, FL and FS fruits, though rich in other valuable phytochemicals, may have developed under different light spectra [9].

One of the most relevant results of our study is that it is possible to associate the upregulation or downregulation of key polyphenols extremely relevant for human health with a specific light-quality growth regime. For instance, tomato plants grown under RB light will produce fruits richer in quercetin and its derivatives (Quercetin Rutinoside, Quercetin-3-O-Galactoside, and Quercetin Acetylhexoside) [13]. These molecules have a higher free radical scavenging activity, modulate pro-inflammatory cytokines, and inhibit cancer cell proliferation. In particular, Rutin and Quercetin Rutinoside have been associated with vascular protection, reducing capillary fragility and permeability, and preventing hypertension, hypercholesterolemia, and cardiovascular diseases (Table S1) [33,34]. Several pieces of evidence have demonstrated that Rutin production, such as chlorogenic and caffeic acid, is stimulated by pure blue light (480 nm) or mixed with UV-B (660 nm) [10,29,35] or red and green wavelengths [36]. Plant growth under RB light also enhances chlorogenic acid and caffeoylquinic acid derivates as well as Apigenin and Apigenin-8-C-Glucoside, with strong antioxidant anticancer and anti-inflammatory properties. Chlorogenic acid has been associated with glucose metabolism regulation as a promising compound in type 2 diabetes prevention (Table S1) [37], while Apigenin-8-C-glucoside has been demonstrated to induce apoptosis in tumor cells without affecting healthy cells (Table S1) [38]. On the other hand, plant growth under full spectra (FS) increases the content of different polyphenols, such as Epicatechin, Catechin, and Ferulic Acid, associated with cardiovascular protection and cognitive benefits (Table S1) [3], and Resveratrol with wide-ranging known effects, including anticancer, cardioprotective, anti-aging, and neuroprotective (Table S1) [23,39,40]. Our data confirm previous studies in which has been evidenced that blue light may have favored the synthesis of these compounds [41]. However, it may be argued that in tomato fruits that are exposed to an elevated % of blue light, it is the combination of blue light with red or far-red that makes the difference, since the amount of these polyphenols is higher in FS than in RB light regime.

An interesting observation is that among the several polyphenols identified in RB, FL, and FS tomatoes, are flavonoids with direct antiviral activity against SARS-CoV-2 (Figure S2) [42], due to their ability to interfere with key steps in the viral life cycle and modulate host immune responses. In this context, tomatoes enriched in these bioactive molecules may represent a valuable dietary strategy for prevention or support during viral infections. More specifically, Quercetin and Rutin, which are more abundant in RB and FS fruits, have emerged as the most studied flavonoids with direct antiviral activity against SARS-CoV-2. Furthermore, Resveratrol, Epicatechin, and Catechin enhanced in FS fruits also help mitigate virus-induced oxidative stress (Table S1) [42]. However, it must be considered that all potential COVID-19-related benefits of these compounds should be further supported by dedicated in vitro and in vivo studies, along with investigations into their bioavailability.

While the physiological benefits of specific light spectra are clear, their practical application must also consider economic feasibility, particularly in commercial horticulture. LEDs provide a cool, energy-efficient, and easily adjustable lighting solution, offering significant advantages over traditional field conditions. Field cultivation often results in the excessive use of soil, water, fertilizers, and pesticides to manage biotic stress. In contrast, LEDs can enhance plant yield and improve biochemical composition, acting as ‘natural fertilizers’ that reduce the need for chemical inputs and lower resource consumption, thus promoting sustainable production.

Red-blue (RB) LED systems are commonly used for their high energy efficiency and lower operational costs. These specific wavelengths are highly effective at driving photosynthesis, making the RB light regime ideal for maximizing biomass with minimal energy loss. In contrast, full-spectrum (FS) lighting, even if more expensive due to its broader wavelength output and higher energy requirements, promotes the synthesis of a wider range of bioactive compounds. 

For high-value crops, particularly those cultivated for nutraceutical or pharmaceutical applications, the enhanced accumulation of health-promoting compounds can justify the additional investment in an FS regime, which may offer a favorable long-term cost–benefit balance.

The Microtom cultivar, a dwarf tomato variety, offers the advantage of high yield in limited space compared to traditional varieties. This property makes Microtom ideal for controlled environments such as vertical farms, small businesses, or even home gardens where the cultivation volume is limited. LED lighting finds large application in these settings and is already widely adopted, enhancing the accessibility and practical relevance of our study, even for small-scale or homemade chambers.

In conclusion, although our study focused on a single cultivar in controlled conditions, future research should enlarge to include more cultivars and large-scale validation trials. Techno-economic analyses are also needed to optimize lighting strategies for productivity and sustainability. Additionally, exploring the antioxidant properties in vitro cultures could open a new scenario for applied research.

## 4. Materials and Methods

### 4.1. Plant Material, Experimental Set-Up

Dry seeds of *Solanum lycopersicum* L. cv ‘Microtom’, furnished by Holland OnlineVof (Amsterdam, The Netherlands), from the same production batch, were sown in 1.2 L pots filled with soil (86% peat, 9% sand, 3% quartz sand, and 2% perlite) and stored in the dark until germination. At 15 days after sowing (DAS), seedlings were distributed under three different light quality regimes (LQ), with 5 pots per light treatment: white fluorescent (FL), full-spectrum (FS), and red-blue (RB) [Figure S2]. The FL regime was obtained by combining fluorescent tubes (LumiluxL36W/640 and L36W/830, Osram, München, Germany), while FS and RB were provided by arranging light emitting diodes (LEDs) (LedMarket Ltd., Plovdiv, Bulgaria). More specifically, FS was obtained by using far-red, red, yellow, green, blue, UV-A, and white light, while RB was obtained by mixing red and blue light in a ratio of R/B 60:40 as described in [20].

The spectral composition of the light regimes was assessed with an SR-3000A spectro-radiometer at a resolution of 10 nm (Macam Photometrics Ltd., Livingston, Scotland, UK). For all LQ regimes, in the growth chamber, the photosynthetic photon flux density (PPFD) was set at 360 µmol photons m^−2^ s^−1^ at the top of the canopy, and the photoperiod was 14 h. The relative air humidity was 60–70% and day/night air temperature 25/20 ± 2 °C. Plants were irrigated weekly to field capacity to replenish water lost by evapotranspiration and fertilized weekly with half-strength Hoagland’s solution [20].

Plants were grown for 120 DAS up to fruit ripening and harvest at the Institute of Plant Physiology and Genetics (IPPG) in Bulgaria and then transferred to the Ecology laboratory at the University of Naples Federico II. Biochemical analyses were performed to evaluate how the modulation of the light spectrum may differently affect the fruit quality. For this purpose, fruits were powdered with liquid nitrogen by using a mortar and a pestle and stored at −80 °C until further biochemical analyses for the determination of soluble proteins, carbohydrates, total carotenoids, lycopene, ascorbic acid concentration, total condensed tannins, total anthocyanins, total flavonoids, total polyphenols, and total antioxidant capacity and activity. Finally, the fruit characterization was completed with a polyphenolic profile determination.

### 4.2. Biochemical Analyses and Polyphenolic Profile

#### 4.2.1. Ferric Reducing Antioxidant Power (FRAP) Assay and DPPH Assay

The total antioxidant capacity of tomato fruits was evaluated performing the ferric-reducing antioxidant power (FRAP) assay, described in [43] and modified by Costanzo et al. [44]. Samples (0.250 g) were extracted in 5 mL of methanol/water solution (60:40, *v*/*v*) for 1 h and centrifuged for 15 min at 14,000 rpm at 4 °C. The extracts were than mixed with the FRAP reagents and incubated in the dark for 1 h. After the reaction, the absorbance was read at 593 nm. Then, the antioxidant capacity was determined using a Trolox (6-hydroxy-2,5,7,8-tetramethylchroman-2-carboxylic acid) standard curve and quantified as µmol Trolox equivalents per gram of fresh weight (µmol TRO eq. g^−1^ FW).

Free radical scavenging activity was assessed by 1,1-diphenyl-2-picrylhydrazyl (DPPH) assay, according to Dudonné et al. [45]. The methanolic extract of sample (67 μL) was mixed with 2 mL of 6 × 10^−5^ M DPPH methanolic solution. The mixture was shaken and incubated at 37 °C for 20 min. The absorbance of the resulting solution was read at 515 nm and converted into the percentage of inhibition of DPPH radicals applying the Equation (1):

Inhibition (%) = [(A_blank_ − A_sample_)/A_blank_] × 100 (1)

where A_blank_ is the absorbance of the blank and As_ample_ is the absorbance of the tested methanolic extract. Trolox was used as the positive control.

#### 4.2.2. Determination of Total Carotenoids, Lycopene, and Ascorbic Acid

The total carotenoids were estimated following the procedure reported by Lichtenthaler [46]. Samples (0.200 g) were extracted in ice-cold 100% acetone and centrifuged (Labofuge GL, HeraeusSepatech, Hanau, Germany) at 5000 rpm for 5 min. Then, the absorbance of the supernatants was measured with a spectrophotometer (Cary 100 UV-VIS, Agilent Technologies, Santa Clara, CA, USA) at 470, 645, and 662 nm. The carotenoid concentration was expressed as mg g^−1^ fresh weight (FW).

The quantification of lycopene concentration was performed as reported by Fish et al. [47] and Davis et al. [48] with slight modifications. Preventively, we prepared a solution of butylated hydroxytoluene (BHT) in acetone at the final concentration of 0.05% (*w*/*v*). Then, 0.500 g of sample was weighed in a beaker and mixed with 20 mL of the mixture hexane/ethanol/0.05% BHT in acetone (*v*/*v*/*v*, 2:1:1). The beaker was covered with an aluminum foil, to protect the sample from light, and placed on a magnetic stirring plate for about 15 min at room temperature. After shaking, 3 mL of deionized water was added to each sample and the shaking step was repeated for 15 min. The sample was then left at room temperature for 5 min to allow phases’ separation. The upper hexane layer was isolated with the help of a pipette and collected in a test tube. The absorbance of the hexane fraction was read in a 1 cm path-length quartz cuvette at 503 nm blanked with hexane. The extinction coefficient of lycopene in hexane (17.2 × 10^4^ M^−1^ cm^−1^) and the lycopene molecular weight (536.9 g mol^−1^) were used to convert the absorbance into lycopene concentration expressed as milligrams per gram of fresh weight (mg g^−1^ FW).

The ascorbic acid (AsA) content was estimated with the Ascorbic Acid Assay Kit (MAK074, Sigma-Aldrich, St. Louis, MO, USA), following the procedure reported by Costanzo et al. [49]. The sample (10 mg) was homogenized in 4 volumes of cold AsA buffer and then centrifuged at 13,000 rpm for 10 min at 4 °C. The liquid fraction was mixed with the AsA assay buffer to a final volume of 120 μL and with the kit reagents to allow the colorimetric coupled enzyme reaction. The colored product developed from the reaction was proportional to the amount of ascorbic acid contained in the sample. The absorbance was read at 570 nm and the concentration of ascorbic acid in the sample was referred to as a standard curve and expressed in ng µL^−1^.

#### 4.2.3. Determination of Soluble Protein and Carbohydrate Content

Total soluble protein content was evaluated as reported by Im et al. [50]. Samples (0.200 g) were treated with 0.2 M potassium phosphate buffer (pH 7.8 + 0.1 mM EDTA) and centrifuged. Then, the supernatant was mixed to the dye reagent, and the absorbance was measured at 595 nm as indicated assay Bradford [51]. The total soluble proteins were quantified by means of a bovine serum albumin (BSA) calibration curve and expressed as mg BSA eq. g^−1^ FW.

The total carbohydrates were quantified with the anthrone method described by Hedge and Hofreiter [52] and slightly modified as in Vitale et al. [8]. Samples (10 mg) were treated with 2.5 N HCl to hydrolyze carbohydrates into simple sugars. Then, the colorimetric reaction was carried out with the anthrone reagent dissolved in ice-cold H_2_SO_4_. The absorbance was measured at 630 nm, and the total carbohydrates were quantified by a glucose calibration curve and expressed as mg glucose equivalents per gram of fresh weight (mg GLU eq. g^−1^ FW).

#### 4.2.4. Determination of Total Condensed Tannins, Anthocyanins, Flavonoids, and Polyphenols

The total condensed tannins were determined following the vanillin assay reported by Sun et al. [53] with slight modifications. Samples (0.200 g) were extracted in methanol solution and centrifuged. Then, 500 µL of extracts were mixed with 1.25 mL of 1% vanillin in methanol (w,v) solution and concentrated H_2_SO_4_ (1:1 *v*/*v*). Samples were incubated for 15 min at room temperature. Then, the absorbance was measured at 500 nm and total condensed tannins were expressed as mg catechin equivalents per gram of fresh weight (mg CAT eq. g^−1^ FW) using a catechin calibration curve.

The anthocyanin concentration was quantified as reported by Mancinelli et al. [54] and Chung et al. [55]. Samples (0.250 g) were extracted in the dark for 24 h using acidified methanol (1% HCl) at 4 °C. Samples were centrifuged, and the absorbance of supernatants was read at 530 and 657 nm. The absorbance values and the extinction coefficient (31.6 M^−1^ cm^−1^) were used to calculate the total anthocyanin content with the following equation: (µmol g^−1^) = [(A530 − 0.33 × A657)/31.6] × [volume (mL)/weight (g)].

Total flavonoid content was estimated following Moulehi et al. [56] and Sun et al. [53]. First, samples (0.200 g) were extracted in methanol at 4 °C and centrifuged. Then, the supernatants (250 µL) were mixed with 5% NaNO_2_ solution (75 µL) and, after 6 min, with 10% AlCl_3_ solution (150 µL) and 1 M NaOH solution (500 µL). The mixture was finally adjusted with distilled water to a final volume of 1.525 mL and the absorbance was measured at 510 nm. The total flavonoid content was calculated using a catechin standard curve and expressed as mg catechin equivalents per gram of fresh weight (mg CAT eq. g^−1^ FW).

The determination of the total polyphenols was performed following Costanzo et al. [44]. Samples (0.200 g) were extracted in methanol at 4 °C and centrifuged for 5 min at 11.000 rpm. Extracts were mixed with 1:1 (*v*/*v*) 10% Folin–Ciocâlteu reagent and shaken. After 3 min, the 700 mM Na_2_CO_3_ solution was added to the mixture in the ratio 5:1 (*v*/*v*). After 2 h of incubation in the darkness, the absorbance of the samples was read at 765 nm. The total polyphenol content was quantified using a gallic acid standard curve and expressed as milligrams of Gallic acid equivalents per gram of fresh weight (mg GA eq. g^−1^ FW).

#### 4.2.5. Polyphenolic Profile

The profile of polyphenols was obtained performing a Liquid Chromatography with tandem mass spectrometry (LC-MS/MS) as reported in Costanzo et al. [9] with some modifications as described below.

*Chemicals and reagents*. All standards were purchased from Sigma-Aldrich. All solutions and solvents were of the highest available purity, suitable for LC–MS analysis and purchased from J. T. Baker (Phillipsburg, NJ, Phillipsburg, NJ, USA).

*Preparation of Standard solutions*. The stock solutions were prepared by adding 1.00 mL aliquots of each analyte to a 10 mL volumetric flask and adjusting the volume with methanol to have a standard solution of each analyte at the concentration of 1000 µg L^−1^. The stock solutions were stored at −20 °C until the analysis. Quantitative analysis was performed by making calibration curves for a set of standard molecules selected for the different classes of analytes under investigation. Standard mixtures were prepared by series dilution: 5.0, 10, 25, 50, and 250 µg L^−1^.

*Sample preparation*. Samples were diluted 1:10 in methanol (*v*/*v*), centrifuged at 10,000 rpm for 10 min and filtered through PTFE 0,45µ syringe filters. The supernatant was transferred into an HPLC autosampler, and 1 µL was analyzed in an LC-MS/MS assay.

*LC–MS/MS instrumentation and conditions*. A total of 1 μL of supernatant was analyzed using an AB-sciex 5500 QTRAP^®^ system with an HPLC system Exion LC™ (Santa Clara, CA, USA). The mobile phase used ware A (0.1% Formic Acid in water) and B (0.1% Formic Acid in acetonitrile), and the flow rate was 0.200 mL min^−1^. The chromatographic gradient was from 20% to 90% in 4 min, held for 2 min, then returned to 20% in 1 min. Tandem mass spectrometry was performed using a Turbo VTM ion source operated in negative ion mode, and the multiple reaction monitoring (MRM) mode was used for the selected analytes.

*Data Processing*. Extracted mass chromatogram peaks of metabolites were integrated using the Skyline software (MacCoss Lab Software, Seattle, WA, USA) [57].

*Quantification of analytes*. The first step of mass spectral analysis consisted of the MRM detection of the analytes individually infused to establish the optimal instrument settings for each compound. Next, standard calibration curves for the selected set of molecules were constructed by plotting peak areas against concentration (µg L^−1^), and linear functions were applied to the calibration curves. The coefficients of determination (R2) were greater than 0.99 for all analytes.

### 4.3. Statistical Analyses and Visualization of the Data

All data analysis was conducted within the R environment v4,5.1. The biochemical markers and HPLC data were collected in two separate tables. High-performance liquid chromatography (HPLC) data were imported, cleaned, and reshaped for analysis. Non-detectable values (“<0.001”) were treated as zeros and redundant entries were removed.

#### 4.3.1. Assumption Testing

To evaluate the suitability of parametric vs. non-parametric methods, each biochemical marker was assessed for normality using the Shapiro–Wilk, Kolmogorov–Smirnov, and Anderson–Darling tests within each treatment group (*n* = 8 per marker). Additionally, homogeneity of variances was tested using the Fligner–Killeen test. Most variables failed at least one assumption in at least one group; therefore, non-parametric approaches were adopted for hypothesis testing and group comparisons.

#### 4.3.2. Biochemical Marker Analysis

Hypothesis testing was conducted using Kruskal–Wallis tests followed by Dunn’s post hoc tests with Holm correction for multiple comparisons. Significance groupings were annotated with letter labels generated using the multcompView package 0.1-10, and results were visualized using faceted boxplots with annotated medians and letters. Additionally, ggstatsplot vv0.13.1 was used to generate between-group comparison plots for each marker, with *p*-values adjusted using the Holm method [Figure S1].

#### 4.3.3. Marker Effect Sizes

Treatment-level median values for each biochemical marker were calculated to assess effect sizes. Fold changes and percentage changes were computed by comparing the highest and lowest median values across treatments. Significant marker-treatment interactions were identified by integrating Dunn’s test results (see assumption testing section), and the strongest effects (*p*.adj < 0.05) were visualized in a heatmap-style tile plot.

#### 4.3.4. Spectral Analysis

Spectral reflectance data were interpolated to a regular 1 nm wavelength grid (350–803 nm) using natural cubic splines, ensuring all interpolated intensities were non-negative. Correlations between smoothed spectra and biochemical markers were computed using Spearman’s method. To control for multiple testing across hundreds of wavelengths–marker comparisons, *p*-values were adjusted using the Benjamini–Hochberg false discovery rate (FDR) method. Significant associations were identified based on adjusted *p*-values (FDR < 0.05), and correlation directionality was visualized across the wavelength spectrum.

#### 4.3.5. HPLC Profiling

Hierarchical clustering of quantified polyphenols was performed using median-centered, scaled values per treatment group. Clustering used Manhattan distances and Ward’s method, and results were visualized as a heatmap.

## 5. Conclusions

The results of our study highlight that by adjusting the spectral composition of light, we can influence the production of specific health-promoting compounds in Microtom fruits. This approach presents exciting opportunities in precision agriculture and biofortification, where strategic light management can enhance the nutritional quality of crops and benefit human health through diet.

We found that tomato plants growth under full-spectrum (FS) light conditions produced fruits with an increased levels of polyphenols, flavonoids, tannins, ascorbic acid, and lycopene compared to berries of plants grown under red-blue (RB) and fluorescent (FL) light conditions. Additionally, plants grown under RB light exhibited fruits with the highest carotenoid levels and antioxidant capacity.

The light environment likely played a key role in modulating the expression of enzymes involved in the phenylpropanoid and flavonoid biosynthesis pathways, ultimately shaping the metabolite profile.

Clustering analysis of different polyphenol classes demonstrated that each tomato type—RB, FL, and FS—contains a unique array of bioactive compounds with significant health benefits. Notably, several polyphenols identified in RB, FL, and FS tomatoes, such as Quercetin, Rutin, and Resveratrol, show promise as complementary therapeutic agents against SARS-CoV-2. The concentrations of these compounds are particularly elevated in tomatoes from plants grown under RB and FS light conditions. While these compounds are not substitutes for vaccines or antiviral drugs, they may act as adjuvants by strengthening host defenses, reducing inflammation, protecting against vascular and lung complications.

Overall, by optimizing light quality to enhance specific polyphenols—especially quercetin, kaempferol, naringenin, and luteolin—tomato crops could be tailored as functional foods with anti-COVID potential, particularly useful in at-risk populations or post-infection recovery phases.

## Figures and Tables

**Table 1:** Strongest and weakest light quality treatments influencing the analyzed markers in tomato fruits.

Marker	Strongest	Weakest	FC
Carotenoids	RB	FL	1.27
Lycopene	FS	FL	1.21
Anthocyanins	FL	RB	1.19
Polyphenols	FS	RB	1.97
Flavonoids	FS	RB	7.66
Tannins	FS	RB	1.31
Ascorbic Acid	FS	RB	1.28
Antioxidant capacity	RB	FS	1.36
Antioxidant activity	FS	FL	1.38
Soluble proteins	FL	FS	1.30
Carbohydrates	FL	FS	2.41

## Data Availability

Data are available from the corresponding authors upon reasonable request.

## Supplementary Materials

The following supporting information can be downloaded at: https://www.mdpi.com/article/10.3390/ijms26125712/s1.
