# Supplementary material for: Harnessing Light Wavelengths to Enrich Health-Promoting Molecules in Tomato Fruits
Source: Int J Mol Sci. 2025 Jun 14;26(12):5712. doi: 10.3390/ijms26125712 (PMC12193516; doi:10.3390/ijms26125712)
Supplement: Supplementary file 1 [file ijms-26-05712-s001.zip › ijms-3676657-supplementary.pdf]

Supplementary Materials

Harnessing Light Wavelengths to Enrich Health-Promoting Molecules in Tomato Fruits

Bruno Hay Mele [1,*,†], Ermenegilda Vitale [1,2,†], Violeta Velikova [3], Tsonko Tsonev [3], Carolina Fontanarosa [4], Michele Spinelli [4], Angela Amoresano [4] and Carmen Arena [1,2]

[1] Department of Biology, University of Naples Federico II, Via Cinthia, 80126 Napoli, Italy; ermenegilda.vitale@unina.it (E.V.); c.arena@unina.it (C.A.)
[2] NBFC—National Biodiversity Future Center, 90133 Palermo, Italy
[3] Institute of Plant Physiology and Genetics, Bulgarian Academy of Sciences, Acad. G. Bonchev Street, Bldg. 21, 1113 Sofia, Bulgaria; violet@bio21.bas.bg (V.V.);
[4] Department of Chemical Sciences, University of Naples Federico II, Via Cinthia, 80126 Napoli, Italy
* Correspondence: bruno.haymele@unina.it
† Co-first authors. These authors contributed equally to this work.

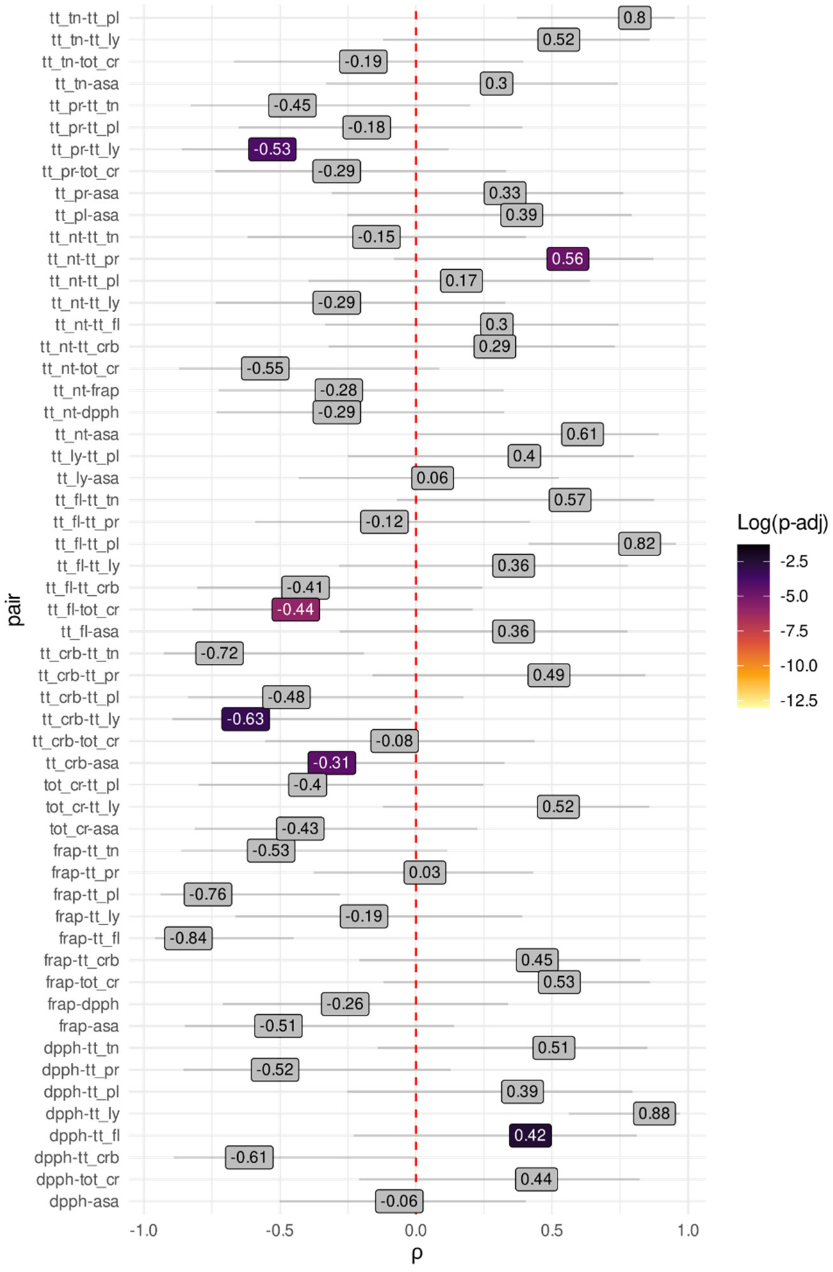

pair
ρ
Log(p-adj)
-2.5
-5.0
-7.5
-10.0
-12.5
-1.0
-0.5
0.0
0.5
1.0
tt_tn-tt_pl 0.8
tt_tn-tt_ly 0.52
tt_tn-tot_cr -0.19
tt_tn-asa 0.3
tt_pr-tt_tn -0.45
tt_pr-tt_pl -0.18
tt_pr-tt_ly -0.53
tt_pr-tot_cr -0.29
tt_pr-asa 0.33
tt_pl-asa 0.39
tt_nt-tt_tn -0.15
tt_nt-tt_pr 0.56
tt_nt-tt_pl 0.17
tt_nt-tt_ly -0.29
tt_nt-tt_fl 0.3
tt_nt-tt_crb 0.29
tt_nt-tot_cr -0.55
tt_nt-frap -0.28
tt_nt-dpph -0.29
tt_nt-asa 0.61
tt_ly-tt_pl 0.4
tt_ly-asa 0.06
tt_fl-tt_tn 0.57
tt_fl-tt_pr -0.12
tt_fl-tt_pl 0.82
tt_fl-tt_ly 0.36
tt_fl-tt_crb -0.41
tt_fl-tot_cr -0.44
tt_fl-asa 0.36
tt_crb-tt_tn -0.72
tt_crb-tt_pr 0.49
tt_crb-tt_pl -0.48
tt_crb-tt_ly -0.63
tt_crb-tot_cr -0.08
tt_crb-asa -0.31
tot_cr-tt_pl -0.4
tot_cr-tt_ly 0.52
tot_cr-asa -0.43
frap-tt_tn -0.53
frap-tt_pr 0.03
frap-tt_pl -0.76
frap-tt_ly -0.19
frap-tt_fl -0.84
frap-tt_crb 0.45
frap-tot_cr 0.53
frap-dpph -0.26
frap-asa -0.51
dpph-tt_tn 0.51
dpph-tt_pr -0.52
dpph-tt_pl 0.39
dpph-tt_ly 0.88
dpph-tt_fl 0.42
dpph-tt_crb -0.61
dpph-tot_cr 0.44
dpph-asa -0.06

Figure S1. Correlation analysis for all pairwise comparisons among dose levels and response markers. Spearman rank correlations (psych::corr.test) with bias-corrected 95 % CIs and Holm-adjusted p-values were calculated for all variable pairs and plotted as point-ranges colored by the log10-transformed adjusted p-value. tot_cr: Total carotenoids; tt_ly: Total lycopene; tt_nt: Total anthocyanins; tt_pl: Total polyphenols; tt_fl: Total flavonoids; tt_tn: Total condensed tannins; asa: Total Ascorbic acid; frap: Total antioxidant capacity; dpph: DPPH radical scavenging activity; tt_pr: Total soluble proteins; tt_crb: Total carbohydrates.

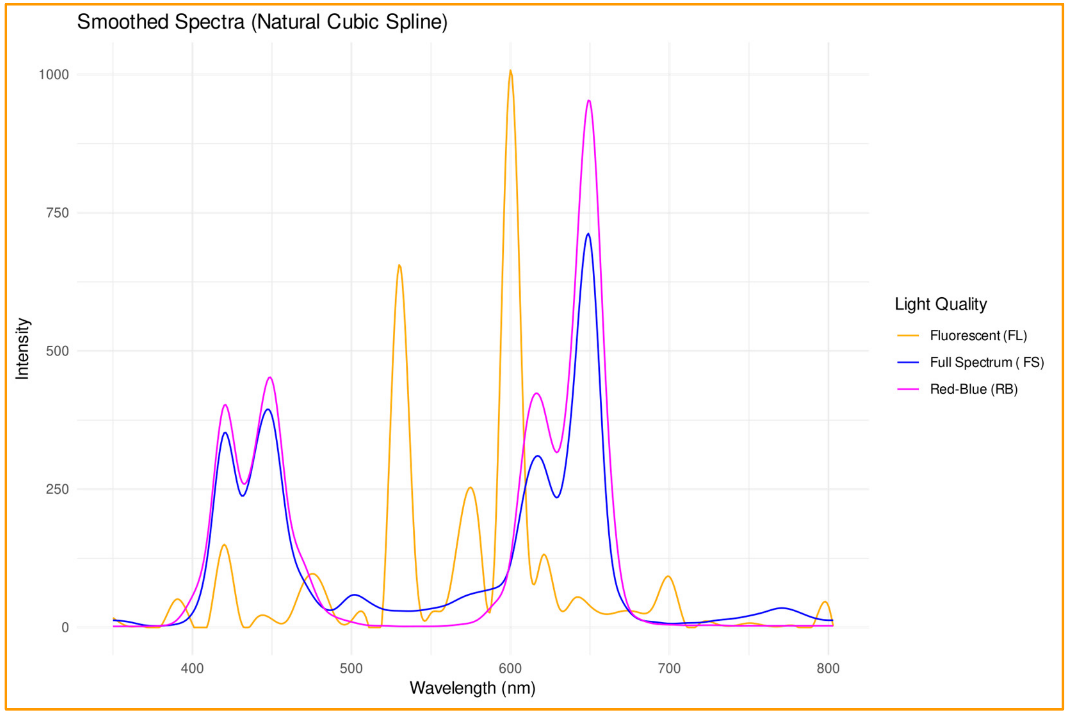

Figure S2. Smoothed spectra of three light qualities (Fluorescent, Full Spectrum, and Red-Blue) using natural cubic spline interpolation. Irradiance Range: 350–800 nm. UV, ultra-violet (350–390 nm); B, Blue (390–500 nm); G, Green (500–560 nm); Y, Yellow (560–600 nm); R, Red (600–700 nm); IR, Far-Red (700–800 nm).

Table S1. Classes of polyphenol compounds as associated to human health benefits

Class	Compound(s)	Health Associations	References
Antioxidant & Anti-inflammatory Effects	3-p-Coumaroylquinic Acid	Potential anti-inflammatory activity via Akt and EGFR inhibition	39220934
	Apigenin	Antioxidant, anti-inflammatory properties.	PMC6472148
Neuroprotective & Cognitive Health	Apigenin	Antioxidant, anti-inflammatory, neuroprotective, cognition-enhancing; potential in Alzheimer's disease treatment/prevention.	PMC6472148
Anti-cancer Properties	Apigenin-8-C-Glucoside	Anti-cancer effects.	27693342
	Apigenin-8-C-Glucoside	Antioxidant, anti-inflammatory effects.	27693342
	Apigenin-8-C-Glucoside	Antioxidant, anti-cancer, anti-inflammatory, neuroprotective effects.	27693342
	Astragalin	Antidiabetic properties; beneficial in diabetes management.	PMC5954929
	Astragalin	Anticancer properties.	PMC5954929
	Astragalin	Antioxidant, anti-inflammatory properties.	PMC5954929
	Astragalin	Neuroprotective, anti-inflammatory, antioxidant properties.	PMC5954929
	Caffeoylquinic Acid Derivative	Antioxidant properties; beneficial effects on human health	28911486
	Catechin	Potential in cancer prevention.	PMC7084675
	Catechin	Antioxidant; modulates gut microbiota and reduces harmful bacteria.	34206736
Cardiovascular Health	Catechin	Antioxidant; supports cardiovascular health by reducing oxidative stress.	34206736
	Chlorogenic Acid	Regulates glucose metabolism; improves insulin resistance; reduces risk of type 2 diabetes.	36576278
	Chlorogenic Acid	Antioxidant, anti-inflammatory activities.	36576278
	Chlorogenic Acid	Regulates glucose and lipid metabolism; reduces risk of type 2 diabetes and cardiovascular diseases.	36576278
	Cis-Resveratrol-3-O-Glucoside	Protective against ethanol-induced brain injury via inhibition of oxidative stress	29708326
	Coumaric Acid	Chemopreventive effects against colon cancer.	PMC6720745
	Coumaric Acid	Antioxidant, anti-inflammatory effects.	16449979
	EC-3-O-Gallate	Inhibits HGF/Met signaling in breast cancer cells	
	EGC Gallate Glucoside	Promotes hair growth and dermal papilla cell proliferation	17092697

;	Epiafzelechin Gallate	Antioxidant; potential in cancer prevention	PMC6247744
	Epicatechin	Enhances oxidative stress status and reduces inflammation in metabolic syndrome	37732427
	Eriodictyol	Antitumor activity.	PMC7752289
	Eriodictyol	Antioxidant, anti-inflammatory properties.	PMC7752289
	Eriodictyol	Neuroprotective, antioxidant, anti-inflammatory effects.	PMC7752289
	Ferulic Acid	Antioxidant; protects against oxidative stress.	34206736
	Ferulic Acid	Antioxidant; mitigates oxidative stress, lowers blood pressure, and supports heart health.	23188120
	Genistin	Decreases risk of osteoporosis and post-menopausal symptoms; anti-cancer, antioxidant, cardioprotective, neuroprotective, hepatoprotective, and antimicrobial activities	32253713
	Hesperetin	Antioxidant; supports heart health by strengthening blood vessels and reducing inflammation.	34206736
Liver Health	Hyperoside	Hepatoprotective; mitigates liver injury and inflammation.	PMC9331122
	Isorhamnetin	Anticancer effects via cell cycle arrest and apoptosis induction	38861855
	Isorhamnetin-3-O-Neohesperidoside	Promotes osteoclast activity; potential role in bone resorption.	PMC7083939
	Isorhoifolin	Antioxidant; anti-inflammatory; potential in neuroprotection	PMC5465813
	Kaempferol	Antioxidant and anti-inflammatory properties; potential in managing various diseases	38731498
	Kaempferol-3-O-Rutinoside	Reduces body temperature by accelerating elimination of IL-6 and TNF-α in fever model	38611918
	Luteolin	Anti-inflammatory and neuroprotective effects	26361743
	Luteolin-6-C-Glucoside (Isoorientin)	Induces apoptosis and decreases invasiveness in pancreatic cancer cells	28003763
;	Luteolin-7-O-Glucoside	Inhibits STAT3 pathway; antiproliferative and antioxidant properties	33525692
	Matairesinol	Anti-inflammatory and antioxidant effects; ameliorates sepsis-mediated brain injury	34705665
	Myricetin	Antioxidant, anticancer, neuroprotective, and cardioprotective effects	34646551
	Myricetin-3-O-Glucoside	Promotes osteogenic differentiation; potential in osteoporosis treatment	34646551
	Naringenin	Antioxidant, anti-inflammatory, anticancer, and cardioprotective properties	30634637
	Naringenin-7-O-Glucoside	Antidiabetic effects; improves insulin resistance and lowers blood glucose levels	30462381

	Naringenin-7-O-Neohesperidoside	Antioxidant; anti-inflammatory; potential in cardiovascular protection	PMC4085189
	Naringin	Hypolipidemic effects; regulates lipid metabolism	35189328
	Nicotiflorin	Anti-inflammatory, antioxidant, neuroprotective, and hepatoprotective effects	35379162
	Orientin	Antioxidant, anti-inflammatory, vasodilatory, and cardioprotective properties	27298620
	Quercetin	Antioxidant, anti-inflammatory, anticancer, and cardioprotective effects	37513932
	Quercetin Acetylhexoside	Antioxidant; anti-inflammatory; potential in neuroprotection	PMC5465813
	Quercetin-3-O-Galactoside	Anti-melanogenesis activity; potential in skin pigmentation disorders	36834475
	Resveratrol	Potential in preventing bowel cancer; protective against type 2 diabetes and aging	29708326
	Tetramethylpyrazine	Neuroprotective; improves cognitive function; reduces inflammation and oxidative stress	PMC8005584
	Theaflavin Digallate	Anti-inflammatory; protects cartilage; potential in osteoarthritis therapy	PMC9286955
	Theaflavin Gallate	Antioxidant; reduces cholesterol absorption; potential in cardiovascular health	PMC8601833
	Theobromine	Neuroprotective; improves cognitive performance; potential in Alzheimer's disease prevention	PMC4335269
